# Oropharyngeal Adverse Events to Drugs and Vaccines: Pharmacovigilance Data From Italy (2019–2021)

**DOI:** 10.1111/odi.15145

**Published:** 2024-10-06

**Authors:** Liberata Sportiello, Gaetano La Mantia, Vera Panzarella, Giuseppe Colella, Simona Potenza, Giacomo Oteri, Laura Sottosanti, Giuseppe Bellavia, Mario Gaio, Alessia Zinzi, Ilaria Morreale, Gaspare Parrinello, Elisabetta Geninatti, Eleonora Marrazzo, Vittorio Fusco, Annalisa Capuano, Giuseppina Campisi

**Affiliations:** ^1^ Campania Regional Centre for Pharmacovigilance and Pharmacoepidemiology University of Campania "Luigi Vanvitelli" Naples Italy; ^2^ Department of Experimental Medicine University of Campania "Luigi Vanvitelli" Naples Italy; ^3^ Unit of Oral Medicine and Dentistry for Fragile Patients, Department of Rehabilitation, Fragility, and Continuity of Care University Hospital Palermo Palermo Italy; ^4^ Department Me.Pre.CC University of Palermo Palermo Italy; ^5^ Department of Biomedical and Dental Sciences and Morphofunctional Imaging University of Messina Messina Italy; ^6^ Oral and Maxillofacial Surgery Unit, Multidisciplinary Department of Medical‐Surgical and Dental Specialties University of Campania "Luigi Vanvitelli" Naples Italy; ^7^ Italian Medicines Agency Rome Italy; ^8^ Department of Drug ASP Agrigento Agrigento Italy; ^9^ Internal Medicine, Pharmacovigilance and Clinical Pharmacology Unit, Department of Promoting Health, Maternal‐Infant, Excellence and Internal and Specialized Medicine (PROMISE) G. D'Alessandro, Sicilian Regional Pharmacovigilance Centre University of Palermo Palermo Italy; ^10^ Department of Promoting Health, Maternal‐Infant, Excellence and Internal and Specialized Medicine (PROMISE) G. D'Alessandro University of Palermo Palermo Italy; ^11^ Piedmont Regional Centre for Pharmacovigilance Turin Italy; ^12^ Oncology Unit Azienda Ospedaliera Di Alessandria SS. Antonio e Biagio e Cesare Arrigo Alessandria Italy; ^13^ Department of Biomedicine, Neuroscience and Advanced Diagnostic University of Palermo Palermo Italy

**Keywords:** database, dentists, oropharyngeal adverse events, pharmacovigilance, spontaneous reporting

## Abstract

**Objectives:**

The aim of this study was to perform a descriptive analysis of oropharyngeal adverse events (AEs) related to drugs and/or vaccines in order to provide useful information for clinicians.

**Methods:**

Data related to three regions of Italy were analyzed from 2019 to 2021 by using the National Pharmacovigilance database.

**Results:**

Among overall 67,384 cases, 2773 (4.1%) reported at least one oropharyngeal AE. Most cases referred to females (71.0%) and adults (70.8%). The majority of cases were reported as not serious (68.4%) and the outcome was mainly positive (73.5%). The cases related to drugs (52.2%) were slightly more than those related to vaccines (47.8%), the latter nearly completely represented by COVID‐19 vaccines. Among 3324 oropharyngeal AEs, the most commonly reported were oropharyngeal conditions (65.9%). The most reported AEs related to vaccines were paresthesia oral and oropharyngeal pain, whereas the most reported AEs related to drugs were throat tightness and angioedema. A marked under‐reporting of osteonecrosis of the jaw (2.9%) was observed, despite this risk was well documented in the same country.

**Conclusions:**

This analysis suggested an under‐reporting of oropharyngeal AEs and the need to better train dentists, dental hygienists, and also general practitioners.

**Abbreviations**
AE: adverse event
AIFA: Agenzia Italiana del Farmaco
ATC: Anatomical Therapeutic Chemical
EMA: European Medicines Agency
EV: EudraVigilance
FDA: Food and Drug Administration
GDP: General Dental Practitioners
HCP: Healthcare Professional
ICSR: Individual Case Safety Reports
IME: Important Medical Event
LLT: Low‐Level Term
MedDRA: Medical Dictionary for Regulatory Activities
MRONJ: medication‐related osteonecrosis of the jaw
O‐AE: Oropharyngeal Adverse Event
PT: Preferred Term
QPPV: Qualified Persons for Pharmacovigilance
RNF: Rete Nazionale di Farmacovigilanza
SMQ: Standardized MedDRA Query
SOC: System Organ Class

## 1 Introduction

Adverse events (AEs) related to drugs and vaccines in oropharyngeal area (*Oropharyngeal Adverse Events* [O‐AEs]) have been reported after the use of several therapeutic classes, such as chemotherapies, anxiolytics and hypoglycemic agents, and more recent drugs belonging to the new “targeted” therapies and/or biological agents for the treatment of numerous oncological, auto‐immune, inflammatory, and rheumatological diseases (Yuan and Woo 2020). However, it is well known that also during dental treatments, it is often necessary to prescribe medications to relieve dental pain and reduce inflammation or infection, potentially inducing O‐AEs.

If not correctly diagnosed and treated, these events at the oropharyngeal area could be responsible for a deterioration in the quality of life of patients (in most cases elderly and/or with oncological diseases) resulting in the withdrawal of therapy and/or (very rarely) in the *exitus* of the patient (Yuan and Woo 2015). However, it is often difficult to establish a clear causal relationship between the administration of a drug and the occurrence of an AE in the oropharyngeal area, due to the multiplicity of involved classes of drugs and other substances (e.g., dental materials and foods), the variety of affected tissues and functions, the type of induced lesions and disturbances (Lo Russo et al. 2012). Moreover, O‐AEs sometimes have a long latency with occurrence also after many years from drug therapy suspension (e.g., osteonecrosis of the jaw) or with a slow development during the prolonged use of a drug (e.g., mucositis). According to this, O‐AEs are poorly reported in the literature and in the spontaneous reporting systems (Ellefsen et al. 2023). Some studies have shown that healthcare professionals may not always report AEs for various reasons, such as having a lack of knowledge or time (Li et al. 2022; Potlog Shchory et al. 2020). The lack of knowledge regarding AE reporting is an issue that needs to be principally addressed during healthcare professionals' training. A recent cross‐sectional study revealed that, in Croatia, pharmacy students outperformed dentistry and medicine students significantly in terms of their knowledge. Among the participants, 92.2% of the pharmacy students, 21.8% of the dentistry students, and 70.8% of the medical students were aware of the importance of patient involvement in reporting AEs (Seselja Perisin et al. 2021). Nevertheless, most of the students expressed their belief that pharmacovigilance was inadequately covered in their study programs (La Mantia et al. 2023; Seselja Perisin et al. 2021). The knowledge of importance of pharmacovigilance and the procedures on the spontaneous reporting of suspected adverse drug reactions are the core competencies that healthcare students should acquire during their studies. Despite no standard exists for teaching pharmacovigilance, at Italian universities medical, pharmacy, dentistry, and nursing students have been educated and trained in this field for more than a decade. Initially, the pharmacovigilance was a part of pharmacology program but today is often placed also as a dedicated program. Therefore, in line with the current European pharmacovigilance dispositions, healthcare professionals (but also patients/citizens) should be aware that they can independently report suspected AEs related to drugs.

In the last decades, great breakthroughs have been made because of the intensive efforts not only by regulatory agencies (e.g., European Medicines Agency [EMA] and Food and Drug Administration [FDA]) but also by all stakeholders in the field of Pharmacovigilance (European Medicines Agency [EMA] 2023b). In Italy, the National Competent Authority (*Agenzia Italiana del Farmaco* [AIFA]) promoted many pharmacovigilance projects with the support of Regional Centers of Pharmacovigilance and of many willing healthcare professionals with expertise in pharmacovigilance and pharmacoepidemiology (Parretta, Sottosanti, et al. 2014). Positive results were obtained by several professional categories, such as pharmacists (Parretta, Rafaniello, et al. 2014), but other healthcare figures remain reluctant, and thus, their role is substantially unexplored. As confirmation of this, at the international and national level, the spontaneous reporting systems are affected by a very low reporting of AEs involving the oropharyngeal area by healthcare professionals, including general practitioners and specialists such as dentists (the latter < 1%; Yip, Radford, and Brown 2013). As a part of the drug safety post‐marketing monitoring system, these healthcare professionals have the duty to report information about AEs that they detect, even if a causal association is uncertain.

Considering the weak attitude of these healthcare professionals in reporting AEs, herein we choose to describe the O‐AEs related to drugs and/or vaccines reported in three large regions of Italy (one of the North—Piedmont, and two of the South—Sicily and Campania) with around 14,869,654 million inhabitants (mean value in the time range 2019–2021, equal to about a quarter of the general population; Italian National Institute of Statistics [Istat] 2023) by using data from the Italian Pharmacovigilance database.

## 2 Methods

### 2.1 Data Source

The RNF (*Rete Nazionale di Farmacovigilanza*) is the Italian database designed for collecting Individual Case Safety Reports (ICSRs) of suspected AEs related to drugs or vaccines, managed by AIFA since 2001. These reports are used for monitoring medicines and vaccine safety after their authorization. The RNF database is directly connected with the EudraVigilance (EV) database of EMA. A special section of AIFA website (https://servizionline.aifa.gov.it/) provides a limited access to drug safety information derived from ICSRs recorded in this database only to the stakeholders involved, such as AIFA, Health Ministry, Italian National Institute of Health, Regional medicine regulatory authorities, Regional Centers of Pharmacovigilance, Qualified Persons for Pharmacovigilance (QPPV) identified in public health settings and Marketing Authorization Holders (Italian Medicines Agency [AIFA] 2023).

From this website, ICSRs can be retrieved individually or in an aggregate manner with an advanced research applying several filters on: origin (from EV, non‐from EV), date of data entry, suspected drug/vaccine, single AE, or groups of AEs, age group (reported in years or months): fetus, 0–1 month, 2 months–2 years, 3–11 years, 12–17 years, 18–64 years, more than 65 years; primary source qualification (physician, hospital doctor, general practitioner, pediatric specialist, pharmacist, other HealthCare Professional‐HCP, nurse, lawyer, patient/non‐HCP), region, and health structure. In aggregate form, all information is reported in a unique Excel file.

ICSRs contain the following information: origin (from EV, non‐from EV), date of data entry, patient sex (female, male, not specified), age (reported in years or months), primary source qualification, adverse reaction list, suspect/interacting and concomitant drug list, outcome, and seriousness. The outcome is categorized as recovered/resolved, recovering/resolving, not recovered/not resolved, fatal, recovered/resolved with sequelae, unknown. Moreover, each case can be classified as not serious or serious. The sub‐criteria for seriousness are the following: results in death, life threatening, congenital anomaly, disabling, caused/prolonged hospitalization. Besides them, when an AE is not covered by these seriousness criteria, but a reporter considered it as serious or it is included in the EMA Important Medical Event (IME) list, it is classified into the criterion “Other Medically Important Condition.” This IME list aims to facilitate the classification of AEs for pharmacovigilance activities in the EU (EMA 2023a). RNF uses the Medical Dictionary for Regulatory Activities' (MedDRA; the internationally standardized medical terminology) coding system for each sign, symptom, or diagnosis of AEs.

MedDRA is a hierarchical system starting with a very general level (the System Organ Classes [SOCs]) and ending with the more detailed level (preferred term [PT]), which is in turn divided into the most specific level, namely, low‐level term (LLT) (Medical Dictionary for Regulatory Activities [MedDRA] 2023b). Each ICSR could include more than one AE codified with MedDRA and more than one suspected drug/vaccine. Standardized MedDRA Queries (SMQs) are validated standard sets of MedDRA terms (typically PTs) related to a variety of safety topics of regulatory interest (e.g., Oropharyngeal disorders, severe cutaneous adverse reactions, and anaphylactic reactions). Some SMQs are a simple set of PTs, whereas other SMQs are hierarchical containing subordinate SMQs (MedDRA 2023a).

### 2.2 Data Retrieval

We searched the RNF database for all ICSRs deriving from the three regions (Piedmont, Campania, and Sicily) involved in the study by using the advanced research function for the period from January 1, 2019, to December 32, 2021.

We performed a procedure on the basis of two steps. Firstly, we obtained an intermediate dataset from RNF by conducting an automated selection of all ICSRs for each Region (with the collaboration of each own Regional Center of Pharmacovigilance) in the considered period. Secondly, we obtained a final dataset selecting only the ICSRs with at least one AE related to the oropharyngeal area for each region. This selection was performed by using two modified MedDRA‐SMQs (version 26.0; MedDRA SMQs 2023): “Osteonecrosis” and “Oropharyngeal disorders.” The latter also included the following five sub‐SMQs: “Oropharyngeal neoplasms,” “Oropharyngeal infections,” “Oropharyngeal allergic conditions,” “Gingival disorders,” and “Oropharyngeal conditions (excluding neoplasms, infections, and allergies)” (MedDRA‐SMQ‐version 26.0). The modification (restriction) of the total PTs list deriving from these SMQs was performed by two independent experts on the basis of their competence in dentistry. This modified PTs list is reported in Table S1. Finally, we shared and united all data from each region.

### 2.3 Data Analyses

The final dataset was built using the software Microsoft Excel. Categorical variables were expressed as percentages. We focused our descriptive analyses only on O‐AEs reported in the selected ICSRs. Therefore, from each ICSR we excluded all the other AEs (PTs) not reported in our modified PTs list deriving from the prespecified SMQs.

All selected ICSRs were categorized for total number of reports, total number of PTs of interest, sex, age, seriousness, outcome, primary source qualification, and type of suspected or concomitant drugs. In each category, we indicated “not specified” if the information was not provided. Moreover, we categorized all PTs into six groups according to the above‐considered SMQs. All PTs classified in these six groups are shown in Table S2.

### 2.4 Compliance With Ethical Standards

Safety data deriving from the spontaneous reporting system are anonymous and follow ethical standards; therefore, no further ethical measure was required.

## 3 Results

**FIGURE 1:**
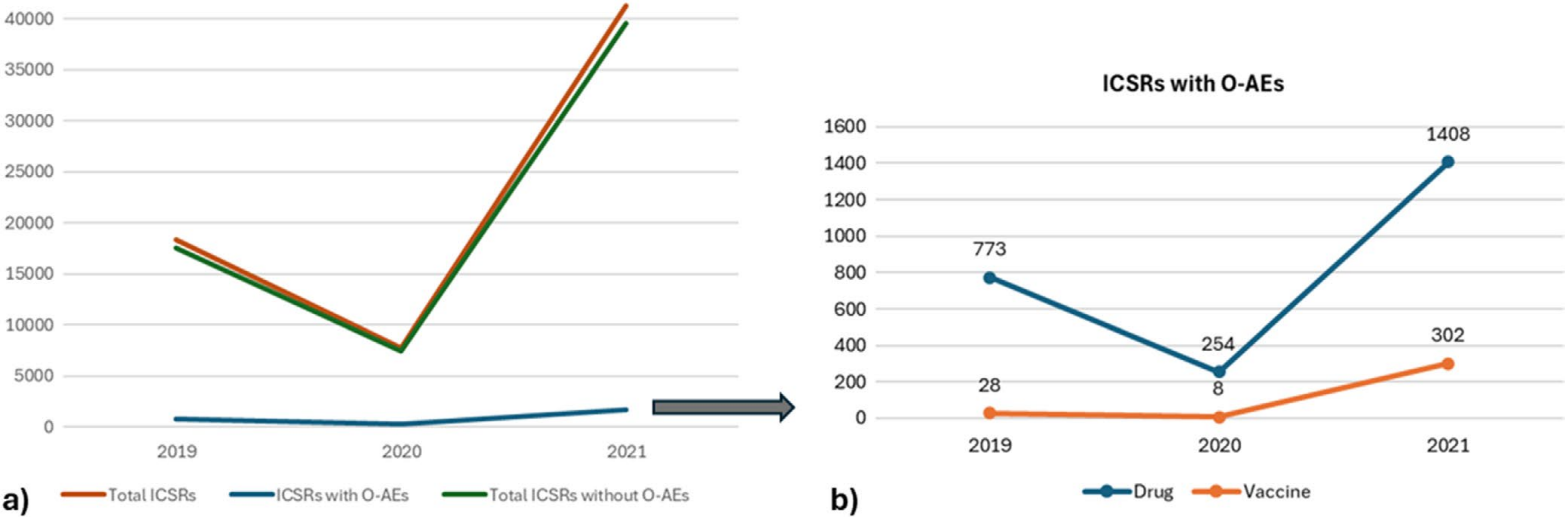
(a) Trend of the total number of Individual Case Safety Reports (ICSRs) and ICSRs with O‐AEs or without O‐AEs from 2019 to 2021. (b) Frequency of ICSRs containing at least one O‐AE stratified by drug and vaccine from 2019 to 2021. ICSR, Individual Case Safety Report; O‐AE, oral adverse event.

In the 3‐year study period (2019–2021), a total of 67,384 ICSRs were retrieved from RNF: 24,764 for Piedmont, 30,509 for Campania, and 12,111 for Sicily (Table 1). Among these ICSRs, 2773 fulfilled the selection criterion on the presence of at least one AE involving the oropharyngeal area. These selected cases represented 4.1% of the total ICSRs deriving from the involved three regions. In the considered period, the overall reporting contribution was of about 42.0%, 38.4%, and 19.5% by Campania, Piedmont, and Sicily, respectively. However, the rate of O‐AE‐related ICSRs in each region was similar. As reported in Figure 1a, a nonlinear increase was observed in both overall and O‐AE‐related reporting trends because there was a negative peak in 2020 (*n* = 7752 and *n* = 262, respectively). The annual counts of the ICSRs reported during the period 2019–2021 are shown in Figure 1b. The majority of ICSRs reported in 2019 were related to drugs (*n* = 773; 96.5%), whereas the remaining cases are related to vaccines (*n* = 28; 3.5%). Similarly, in 2020 the stratification for drugs and vaccines is as follows: (*n* = 254; 96.9%) and (*n* = 8; 3.1%). On the contrary, the majority of ICSRs in 2021 were related to vaccines (*n* = 1408; 82.3%), whereas only 17.7% referred to drugs (*n* = 302).

**TABLE 1:** Characteristics of Individual Case Safety Reports (ICSRs) containing at least one oropharyngeal adverse event (O‐AE).

Characteristics	Level	Piedmont region	Campania region	Sicily region	Total (%)
ICSRs	Number	24,764	30,509	12,111	67,384
ICSRs with O‐AEs^a^	Number	1066 (4.3)	1165 (3.8)	542 (4.5)	**2773 (4.1)**
O‐AEs^a^	Total number	1277	1379	668	**3324**
O‐AEs^a^ per ICSR	Mean	1.20	1.18	1.23	**1.20**
Sex^b^	Male	244 (22.9)	389 (33.4)	157 (29.0)	790 (28.5)
	Female	816 (76.5)	770 (66.1)	382 (70.5)	1968 (71.0)
	Not specified	6 (0.6)	6 (0.5)	3 (0.4)	15 (0.5)
Age group^b^	< 17 years	60 (5.6)	127 (10.9)	15 (2.8)	202 (7.3)
	**18–64 years**	797 (74.8)	780 (67.0)	385 (71.0)	**1962 (70.8)**
	> 65 years	202 (18.9)	246 (21.1)	129 (23.8)	577 (20.8)
	Not specified	7 (0.7)	12 (1.0)	13 (2.4)	32 (1.2)
Seriousness^b^	**Not serious**	734 (68.9)	836 (71.8)	328 (60.5)	**1898 (68.4)**
	Serious	327 (30.7)	324 (27.8)	212 (39.1)	863 (31.1)
	Other IME	171 (52.3)	176 (54.3)	114 (53.8)	461 (53.4)
	Hospitalization	122 (37.3)	126 (38.9)	47 (22.2)	295 (34.2)
	Life threatening	17 (5.2)	14 (4.3)	18 (8.5)	49 (5.7)
	Disabling	7 (2.1)	3 (0.9)	31 (14.6)	41 (4.8)
	Results in death	10 (3.1)	5 (1.5)	2 (0.9)	17 (2.0)
	Congenital anomaly	—	—	—	—
	Not specified	5 (0.5)	5 (0.4)	2 (0.4)	12 (0.4)
Outcome^b^	**Recovered/Resolved**	485 (45.5)	502 (43.1)	211 (38.9)	**1198 (43.2)**
	Recovering/Resolving	290 (27.2)	441 (37.9)	110 (20.3)	841 (30.3)
	Not Recovered/Resolved	169 (15.9)	120 (10.3)	133 (24.5)	422 (15.2)
	Recovered with sequelae	20 (1.9)	16 (1.4)	17 (3.1)	53 (1.9)
	Fatal	10 (0.9)	5 (0.4)	2 (0.4)	17 (0.6)
	Unknown	92 (8.6)	81 (7.0)	69 (12.7)	242 (8.7)
Source^b^	**Physician/doctor**	432 (40.5)	687 (59.0)	372 (68.6)	**1491 (53.8)**
	Pharmacist	291 (27.3)	112 (9.6)	63 (11.6)	466 (16.8)
	Other HCP	115 (10.8)	178 (15.3)	11 (2.0)	304 (11.0)
	Lawyer	—	—	1 (0.2)	1 (0.0)
	Patient/non‐HCP	228 (21.4)	188 (16.1)	95 (17.5)	511 (18.4)
Suspected medicine	**Drug**	378 (33.1)	1016 (67.9)	278 (49.4)	**1672 (52.2)**
	Vaccine	763 (66.9)	480 (32.1)	285 (50.6)	1528 (47.8)

Abbreviations: HCP, healthcare professional; ICSR, Individual Case Safety Report; IME, Important Medical Event.

^a^ O‐AEs codified with PTs of interest reported in our modified PTs list (Table S1).

^b^ Of total received ICSRs.

### 3.1 Characteristics of the Selected Individual Case Safety Reports

Most ICSRs referred to patients aged between 18 and 64 years (*n* = 1962; 70.8%) and female (*n* = 1968; 71.0%; Table 1). The majority of ICSRs were reported as not serious (*n* = 1898; 68.4%). A total of 863 (31.1%) safety cases met seriousness criteria. The criterion most reported was other medically important condition (*n* = 461; 53.4%), followed by caused/prolonged hospitalization (*n* = 295; 34.2%), life threatening (*n* = 49; 5.7%), disabling (*n* = 41; 4.8%), and results in death (*n* = 17; 2.0%). Only for the 0.4% (*n* = 12), this information was not available.

The outcome was reported for 2531 (91.3%) ICSRs and was mainly positive (recovered/resolved or recovering/resolving; *n* = 2039; 73.5%). Only a little percentage of safety cases (*n* = 17; 0.6%) had a fatal outcome. Specifically, only three cases occurred in over‐70‐year patients exposed to COVID‐19 vaccines (mRNA‐Pfizer).

The physician/doctor was the most frequent reporting source of the selected ICSRs (*n* = 1491; 53.8%), followed by the patient/non‐HCP (*n* = 511; 18.4%) and the pharmacist (*n* = 466; 16.8%). The ICSRs related to drugs (*n* = 1672; 52.2%) were slightly more than those related to vaccines (*n* = 1528; 47.8%). All characteristics of ICSRs for each Region are reported in Table 1.

### 3.2 Characteristics of Oropharyngeal Adverse Events

The overall 2773 ICSRs accounted for a total of 3324 O‐AEs reported (median = 1.2 AE per ICSR). Taking account that the distribution of AEs was categorized by SMQs, the most common SMQ was “Oropharyngeal conditions (excluding neoplasms, infections and allergies)” (*n* = 2192; 65.9%), followed by “Oropharyngeal allergic conditions” (*n* = 453; 13.6%), “Osteonecrosis” (*n* = 362; 10.9%), “Oropharyngeal infections” (*n* = 207; 6.2%), “Gingival disorders” (*n* = 106; 3.2%), and “Oropharyngeal neoplasms” (*n* = 4; 0.1%; Table 2). Analyzing data by each SMQ, more than the half of AEs (52.8%) included in the first SMQ were oropharyngeal pain, throat tightness, paresthesia oral, dysphagia, and dry mouth. The 85.4% of the AEs related to the second SMQ “Oropharyngeal allergic conditions” were angioedema, tongue edema, swelling of tongue, palatal edema, and oropharyngeal edema. The first PT in the third SMQ “Osteonecrosis” reported was bone pain, which alone covered the 48.9% of the AEs related to this SMQ. Although the other SMQs were involved for less of 10%, “Oropharyngeal infections” were mainly characterized by oral herpes (52.7%), “Gingival disorders” by gingival bleeding (63.2%) and “Oropharyngeal neoplasms” by leukoplakia oral (50.0%).

**TABLE 2:** Distribution of the first five preferred terms (PTs) for each Standardized MedDRA Query (SMQ) in the Individual Case Safety Reports (ICSRs) containing at least one oropharyngeal adverse event (O‐AE).

	Vaccines	Drugs	Overall
Standardized MedDRA Query			
Oropharyngeal conditions (excluding neoplasms, infections, and allergies)	1630 (74.4)	562 (25.6)	2192 (100.0)
Oropharyngeal pain	**224 (13.7)**	52 (9.2)	276 (12.6)
Throat tightness	142 (8.7)	**124 (22.0)**	266 (12.1)
Paresthesia oral	**208 (12.7)**	48 (8.5)	256 (11.7)
Dysphagia	109 (6.7)	**72 (12.8)**	181 (8.3)
Dry mouth	102 (6.2)	**74 (13.1)**	177 (8.1)
Oropharyngeal allergic conditions	213 (47.0)	240 (53.0)	453 (100.0)
Angioedema	56 (26.3)	**110 (45.8)**	166 (36.6)
Tongue edema	33 (15.5)	**41 (17.0)**	74 (16.3)
Swelling of tongue	**71 (33.3)**	0 (—)	71 (15.7)
Palatal edema	**36 (16.9)**	15 (6.2)	47 (10.4)
Oropharyngeal edema	**29 (13.6)**	0 (—)	29 (6.4)
Osteonecrosis	188 (51.9)	174 (48.1)	362 (100.0)
Bone pain	**155 (82.4)**	22 (12.6)	177 (48.9)
Osteonecrosis of jaw	0 (—)	**95 (54.6)**	95 (26.2)
Osteonecrosis	0 (—)	28 (16.0)	28 (7.7)
Jaw pain	**13 (6.9)**	7 (4.0)	20 (5.5)
Dental abscess	8 (4.2)	**9 (5.1)**	17 (4.7)
Oropharyngeal infections	118 (90.8)	89 (9.2)	207 (100.0)
Oral herpes	**75 (63.5)**	34 (38.2)	109 (52.7)
Gingivitis	7 (5.9)	**22 (24.7)**	29 (14.0)
Pharyngitis	**19 (16.1)**	8 (8.9)	27 (13.0)
Oral candidiasis	2 (1.7)	**12 (13.4)**	14 (6.8)
Tonsillitis	3 (50.0)	3 (50.0)	6 (2.9)
Gingival disorders	63 (59.4)	43 (40.6)	106 (100.0)
Gingival bleeding	39 (61.9)	**28 (65.1)**	67 (63.2)
Gingival pain	4 (6.3)	**6 (13.9)**	10 (9.4)
Noninfective gingivitis	4 (6.3)	**5 (11.6)**	9 (8.5)
Gingival hypertrophy	2 (3.1)	**3 (6.9)**	5 (4.7)
Gingival discomfort	**4 (6.3)**	0 (—)	4 (3.8)
Oropharyngeal neoplasms	1 (25.0)	3 (75.0)	4 (100.0)
Leukoplakia oral	0 (—)	**2 (66.6)**	2 (50.0)
Oral neoplasm	0 (—)	**1 (33.3)**	1 (25.0)
Oral papilloma	**1 (4.0)**	0 (—)	1 (25.0)

*Note:* See Table S2 for all PTs divided into these six SMQs.

**FIGURE 2:**
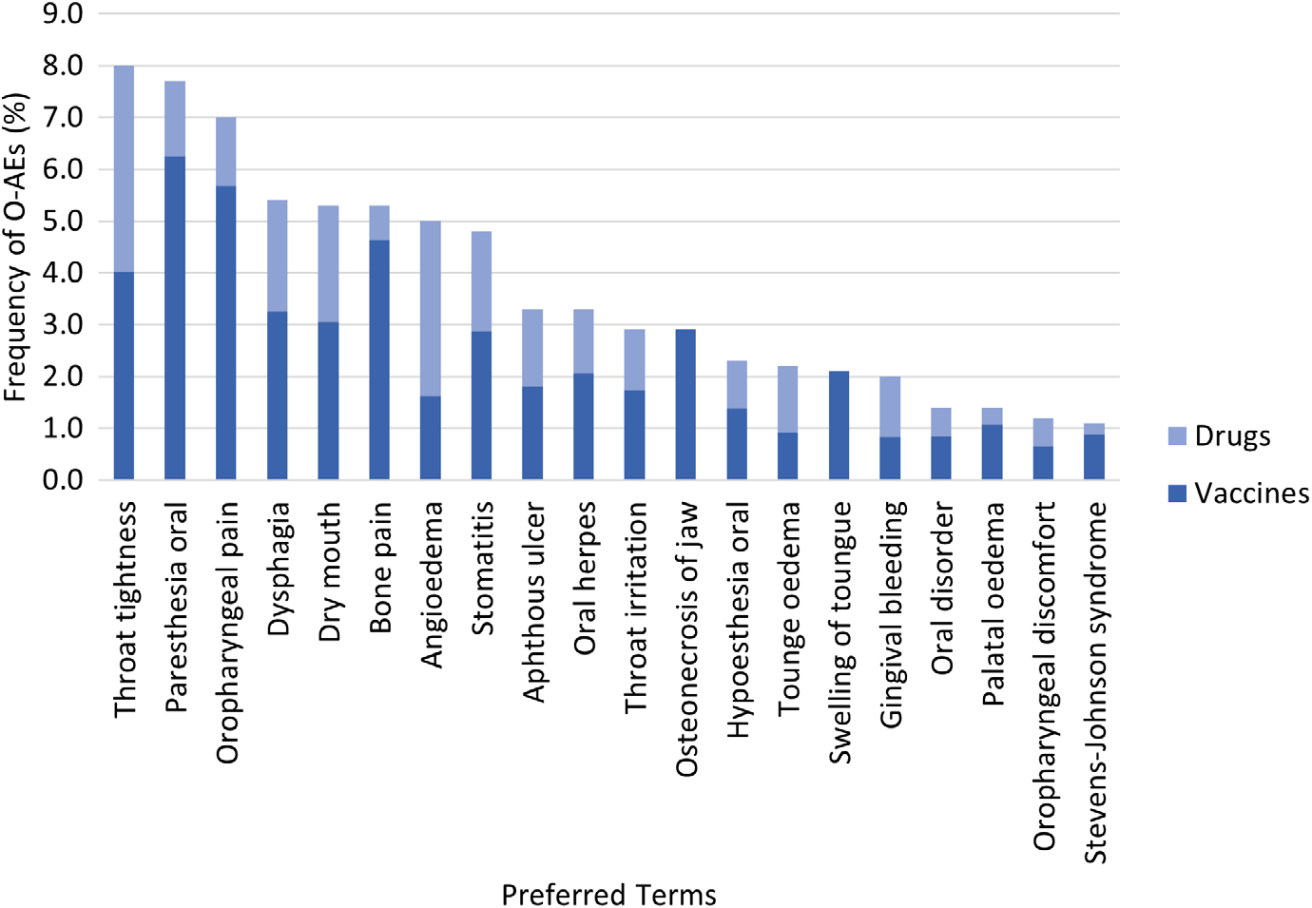
“Top twenty” preferred term (PTs) reported in the Individual Case Safety Reports (ICSRs) containing at least one oropharyngeal adverse event (o‐AEs) from 2009 to 2021.

Specifically, in Figure 2, we reported the first “top twenty” PTs stratified by the type of suspected medicinal products (i.e., vaccine or drug), where the vaccines are completely COVID‐19 vaccines. We observed that vaccines were less reported than drugs in the ICSRs including angioedema, tongue edema, and gingival bleeding (Figure 2). Most of them belonged to oropharyngeal conditions (excluding neoplasms, infections, and allergies) with oropharyngeal pain as the first PT of the SMQ “oropharyngeal disorders” (*n* = 276; 12.6%). See Table S2 for all PTs in each SMQ.

Moreover, we checked whether the most reported O‐AEs were mentioned in the Summary of Product Characteristics (SmPCs) of suspected medicinal products. According to this, we observed a dynamic scenario. No O‐AE is documented in the SmPCs for the most reported vaccines, except for oral paresthesia which is documented for some of the COVID‐19 vaccines. In regard to suspected drugs, the oropharyngeal pain, which is the first most reported O‐AE, was not provided as such in SmPCs of etoposide, which is the first most reported drug related to this event; the same occurred for throat tightness and oxaliplatin or dysphagia and fluorouracil. On the contrary, other most reported O‐AEs are known for the suspected drugs, such as osteonecrosis of jaw and denosumab/zoledronic acid or angioedema and ramipril.

### 3.3 Distribution of Suspected Drugs or Vaccines Reported in the Selected ICSRs

Taking account the distribution of suspected medicines grouped by therapeutic group (first level of Anatomical Therapeutic Chemical [ATC]), we observed that the most common reported level ATC groups were “J—Antiinfectives for systemic use” (*n* = 1734; 54.2%), “L—Antineoplastic and immunomodulating agents” (*n* = 783; 24.5%), “M—Musculo‐skeletal system” (*n* = 272; 8.5%), “N—Nervous system” (*n* = 125; 3.9%), and “B—Blood and blood forming organs” (*n* = 74; 2.3%; Table 3). The other ATC groups individually covered < 2%. The ATC group J was mainly characterized by the second‐level ATC groups “J07‐vaccines” (*n* = 1528, 47.8%) and “J01—antibacterials for systemic use” (*n* = 188; 5.9%); the second ATC group L by “L01—antineoplastic agents” (*n* = 633; 19.8%), and “L04—Immunosuppressants” (*n* = 127; 4.0%; Table 3).

**TABLE 3:** Distribution of suspected medicinal products classified by therapeutics group (second level—Anatomical Therapeutic Chemical, ATC).

ATC—level	*N* (%)
J—Antiinfectives for systemic use	1734 (54.2)
J01 Antibacterials for systemic use	188 (5.9%)
J02 Antimycotics for systemic use	4 (0.1)
J04 Antimycobacterials	1 (0.0)
J05 Antivirals for systemic use	10 (0.3)
J06 Immune sera and immunoglobulins	3 (0.1)
J07 Vaccines	1528 (47.8)
L—Antineoplastic and immunomodulating agents	783 (24.5)
L01 Antineoplastic agents	633 (19.8)
L02 Endocrine therapy	7 (0.2)
L03 Immunostimulants	16 (0.5)
L04 Immunosuppressants	127 (4.0)
M—Musculo‐skeletal system	272 (8.5)
M01 Anti‐inflammatory and antirheumatic products	79 (2.5)
M02 Topical products for joint and muscular pain	14 (0.4)
M03 Muscle relaxants	11 (0.3)
M04 Antigout preparations	23 (0.7)
M05 Drugs for treatment of bone diseases	144 (4.5)
M09 Other drugs for disorders of the musculo‐skeletal system	1 (0.0)
N—Nervous system	125 (3.9)
N01 Anesthetics	1 (0.0)
N02 Analgesics	62 (1.9)
N03 Antiepileptics	16 (0.5)
N04 Antiparkinsonian drugs	1 (0.0)
N05 Psycholeptics	31 (1.0)
N06 Psychoanaleptics	12 (0.4)
N07 Other nervous system drugs	2 (0.1)
B—Blood and blood forming organs	74 (2.3)
B01 Antithrombotic agents	65 (2.0)
B02 Antihemorrhagics	2 (0.1)
B03 Antianemic preparations	6 (0.2)
B05 Blood substitutes and perfusion solutions	1 (0.0)
C—Cardiovascular system	56 (1.8)
C01 Cardiac therapy	10 (0.3)
C02 Antihypertensives	2 (0.1)
C03 Diuretics	1 (0.0)
C04 Peripheral vasodilators	1 (0.0)
C07 Beta blocking agents	4 (0.1)
C08 Calcium channel blockers	3 (0.1)
C09 Agents acting on the renin‐angiotensin system	24 (0.8)
C10 Lipid modifying agents	11 (0.3)
V—Various	49 (1.5)
V01 Allergens	15 (0.5)
V03 All other therapeutic products	7 (0.2)
V08 Contrast media	25 (0.8)
V09 Diagnostic radiopharmaceuticals	1 (0.0)
V10 Therapeutic radiopharmaceuticals	1 (0.0)
A—Alimentary tract and metabolism	40 (1.3)
A01 stomatological preparations	1 (0.0)
A02 drugs for acid‐related disorders	12 (0.4)
A03 drugs for functional gastrointestinal disorders	5 (0.2)
A04 antiemetics and antinauseants	1 (0.0)
A06 Drugs for constipation	3 (0.1)
A07 Antidiarrheals, intestinal anti‐inflammatory/anti‐infective agents	9 (0.3)
A10 Drugs used in diabetes	7 (0.2)
A11 Vitamins	1 (0.0)
A16 Other alimentary tract and metabolism products	1 (0.0)
R—Respiratory system	33 (1.0)
R01 Nasal preparations	1 (0.0)
R02 Throat preparations	3 (0.1)
R03 Drugs for obstructive airway diseases	14 (0.4)
R05 Cough and cold preparations	6 (0.2)
R06 Antihistamines for systemic use	7 (0.2)
R07 Other respiratory system products	2 (0.1)
H—Systemic hormonal preparations, excl. sex hormones and insulins	13 (0.4)
H02 Corticosteroids for systemic use	11 (0.3)
H03 Thyroid therapy	1 (0.0)
H05 Calcium homeostasis	1 (0.0)
D—Dermatologicals	8 (0.3)
D01 Antifungals for dermatological use	2 (0.1)
D08 Antiseptics and disinfectants	1 (0.0)
D10 Anti‐acne preparations	2 (0.1)
D11 Other dermatological preparations	3 (0.1)
G—Genito urinary system and sex hormones	8 (0.3)
G03 Sex hormones and modulators of the genital system	2 (0.1)
G04 Urologicals	6 (0.2)
P—Antiparasitic products, insecticides and repellents	3 (0.1)
P01 Antiprotozoals	2 (0.1)
P02 Anthelmintics	1 (0.0)
S—Sensory organs	
S01 Ophthalmologicals	2 (0.1)
Total	3200

*Note:* The percentage is calculated on the total number of suspected medicines (*n* = 3200). See Table S3 for all suspected drugs/vaccines categorized as second level ATC.

In Table S3, we reported in detail the distribution frequency of suspected medicinal products (as active substances) for each second‐level ATC group reported in the selected ICSRs. The first two suspected products were mRNA and viral vector‐based COVID‐19 vaccines, respectively (*n* = 1203; 37.6% and *n* = 212; 6.6%). The suspected drugs were fluorouracil (*n* = 83; 2.6%), denosumab (*n* = 60; 1.9%), oxaliplatin (*n* = 57; 1.8%), amoxicillin/clavulanic acid (*n* = 51; 1.6%), placlitaxel (*n* = 42; 1.3%), zoledronic acid (*n* = 38; 1.2%), methotrexate (*n* = 37; 1.2%), meningococcal vaccine (*n* = 35; 1.1%), and rituximab (*n* = 34; 1.1). The other medicines covered < 1%.

## 4 Discussion

In our study, we described the O‐AEs related to drugs and/or vaccines by analyzing post‐marketing data from the Pharmacovigilance database of three regions of Italy in a limited period (2019–2021). Although the spontaneous reporting systems are often suffering from incomplete information, we considered all 2773 reports retrieved from the RNF database. The overall and O‐AE‐related spontaneous reporting up‐trends showed a negative peak in 2020, probably mainly referred to the COVID‐19 pandemic and the resulting closure of many activities. On the contrary, the great increase in the reporting in 2021 compared with 2019 was probably related to the same cause (COVID‐19 pandemic) because of a stricter attention also by the media and the public to the administration of the new COVID‐19 vaccines to fight the virus. Similar findings were reported by Zhang et al., who analyzed a dataset of 1,425,371 reports involving 2821 drugs and 7761 AEs, observing a 4.4% decrease in the total number of reports from 2019 to 2020 (Zhang, Sumathipala, and Zitnik 2021).

In a 3‐year period, O‐AEs accounted for approximately 4% of all AEs reported in a part of Italian territory. Also, Ellefsen et al. (2023) showed a similar percentage in the O‐AE reporting (5%) in a longer period (10 years) in Denmark. In our study, in line with the results of this Danish study, the physician/doctor was the most frequent reporting source also in our selected ICSRs (53.8%). Moreover, the patient/non‐HCP and the pharmacist gave a lower but similar contribution in the reporting (18.4% vs. 16.8%). The role of a reporter in referring adverse drug events is different according to his background and competency (Blenkinsopp et al. 2007; Mascolo et al. 2023). In fact, although HCPs (especially the physicians or the medical specialists) can directly or indirectly report a diagnosis or detailed clinical symptoms and signs, non‐HCPs such as patients can only describe the events.

Almost all ICSRs (91.6%) involved patients of adult age and elderly. Moreover, most ICSRs referred to female patients (*n* = 1968; 71.0%). Sex differences in the drug toxicity have been widely discussed over the last decades. Many studies underlined adverse drug events are more common in women than in men (Franconi et al. 2007; Rademaker 2001; Watson et al. 2019) because female sex seems to represent an additional risk factor for AEs and this risk rises with advancing age and with the number of prescribed medicines (Tran et al. 1998). Our finding is also in line with the more recent data derived from the spontaneous reporting systems (Brabete et al. 2022). In fact, Holm et al. investigated how reporting of AEs among adults was related to age and sex by using the Swedish pharmacovigilance database. The results showed that women had higher AE reporting rates than men, with predominance for nonserious cases, in comparison with men who had a higher reporting rate of serious ones (Holm, Ekman, and Jorsäter Blomgren 2017). Moreover, the factors which can promote the spontaneous reporting of AEs have been explored (D'Incau et al. 2014; Lopez‐Gonzalez, Herdeiro, and Figueiras 2009). For example, Figueiras et al. showed that the probability of reporting was double in male physicians compared with female physicians (Figueiras et al. 1999; Holm, Ekman, and Jorsäter Blomgren 2017), whereas other evidence suggested that no difference between genders has been observed regarding factors responsible for the choice to report (Holm, Ekman, and Jorsäter Blomgren 2017).

It is important to consider that AEs can be caused by both immunological and non‐immunological mechanisms, whether related to drugs or vaccines. Immunological mechanisms include hypersensitivity reactions, classified by Gell and Coombs into four types: type I (immediate hypersensitivity), type II (cytotoxic), type III (immune complex‐mediated), and type IV (delayed hypersensitivity) (Gell and Coombs 1963). These reactions can manifest as, for example, Quincke's edema or erythema multiforme in response to drugs or vaccines (Fadul et al. 2022; Yousefian and Khadivi 2023). Non‐immunological mechanisms, on the contrary, can result from direct side effects, such as xerostomia induced by anticholinergic agents or osteonecrosis of the jaw associated with antiresorptive drugs, as well as adverse reactions to vaccines, which may include local or systemic symptoms not mediated by the immune system (Parés‐Badell et al. 2021; Tofé, Bagán, and Bagán 2020). These factors highlight the complexity and variability of responses to drugs and vaccines in patients (Aronson 2003).

In the analyzed period, which included the first year of COVID‐19 vaccines administration (2021), COVID‐19 vaccines (J‐ATC) were the first suspected medicines most reported. Diana Montes‐Grajales et al. suggested that the relevant increase in reports exclusively related to COVID‐19 vaccines within spontaneous reporting databases and in a short period of time can potentially mask certain AEs. This means that external factors can strongly influence the spontaneous reporting of AEs, modifying drug safety analysis. They suggested that the analyses should be accurately corrected for “COVID‐19 factor” (Montes‐Grajales, Garcia‐Serna, and Mestres 2023). Therefore, we cannot exclude that there was a distortion of our data interpretation due to a wide reporting of COVID‐19 vaccines during the period we analyzed.

The appropriate assignment of the seriousness of an AE plays a key role in ensuring that the reporting is suitable to regulatory timelines, which are shorter if the AE is serious (Routray et al. 2020). In contrast with Ellefsen et al. (2023), in our study the rate of serious cases (31.1%) was lower than not serious ones (68.4%). We hypothesized that this finding could depend on the type of drugs involved. Specifically, most of the not serious O‐AEs were related to COVID‐19 vaccines, which were the most reported medicinal products. Ahsanuddin et al. assessed the frequency of otolaryngologic AEs following COVID‐19 vaccination in comparison with other vaccines by using the US national registry (FDA's Vaccine Adverse Event Reporting System, VAERS). In line with our results, they found that few otolaryngologic symptoms were clinically significant (Ahsanuddin et al. 2023). Another study reported O‐AEs (including taste dysfunction, oral mucosal lesions, and salivary gland disorders) in association with COVID‐19 vaccines and these events had a similar distribution in comparison with seasonal influenza vaccines and they seem to be rare (Riad et al. 2022). Riad et al. (2023) conducted a secondary data analysis of all potential O‐AEs reported after COVID‐19 vaccination by using EV database. Although with a low prevalence, the most common O‐AEs were taste‐related, other sensory, and anaphylactic events. In agreement with this, our results showed that the most commonly reported O‐AEs were oropharyngeal conditions (65.9%), including oropharyngeal pain, throat tightness, oral paresthesia, dysphagia, and dry mouth, related to the COVID‐19 vaccines.

Taking account that O‐AEs may be brought on using different medications during dental operations or as side effects or adverse drug–drug interactions from using systemic medications (Ouanounou, Ng, and Chaban 2020), we observed O‐AEs widely documented in literature as various oral soft tissue manifestations or few hard tissue conditions. Among oropharyngeal conditions, AEs can impact the salivary glands, resulting in hyposalivation (lower saliva flow), which causes a dry mouth. This O‐AE is reported in our research in 177 cases; alternatively, they can result in secondary Sjogren's syndrome as a result of drugs such as PD‐1 immune checkpoint inhibitors (Warner et al. 2019), and in our dataset, 11 cases of O‐AE by nivolumab are reported. On the contrary, certain patients may experience excessive salivation (sialorrhea), which can result in more saliva or trouble swallowing as a result of drugs such as antipsychotics, general anesthetics, anticholinesterases, anxiolytics, and others (Teoh, Moses, and McCullough 2019). In our dataset, 33 cases corresponded to the diagnosis of sialorrhea.

When using medications such anticonvulsants, calcium channel blockers, or immunomodulators, certain individuals may undergo tissue oropharyngeal swelling, leading also to gum enlargement (Bharti and Bansal 2013). Others may experience Quincke's edema, a swelling of the oral mucosa that affects the lips, tongue, and in rare cases, the uvula, after taking medications such angiotensin‐converting enzyme (ACE) inhibitors, local anesthetics, and antibiotics (Bakhtiari et al. 2018). These types of O‐AEs are also the most found in our dataset. Additionally, another O‐AE reported in the literature is oral candidosis, causing white and red patches or lesions on the oral mucosa and can be facilitated by antibiotics, corticosteroids, or immunomodulators (Lu 2021), but reported only in 14 cases in our dataset.

Additionally, after using medications such as ACE inhibitors, local anesthetics, aspirin, and others, cheilitis, an inflammation of the lips that manifests as redness, burning, and fissures, may develop (Lugović‐Mihić 2018).

O‐AEs, more rarely, can also result in erythema multiforme, a mucocutaneous disorder that can cause the entire oral mucosa to become covered in red, puffy, and vesicular sores. Some of the pharmaceuticals that can exacerbate this syndrome are antibiotics, anticonvulsants, antiretrovirals, and nonsteroidal anti‐inflammatory drugs (NSAIDs; Aswath and Rakshana 2022), reported in 35 cases in our dataset. Similarly, the fixed drug eruption is rare as being another potential lesion that is defined by the recurrence of a rash or lesion at the same site every time the medication is administered. Among the most often used drugs are anesthetics, antibiotics, antiseptics, mouthwashes, and toothpaste (Gupta 2003).

Further other O‐AEs affecting soft tissues include lupus‐like lesions, which can manifest as mouth ulcers or skin rashes after taking medications such as beta‐blockers, anticonvulsants, chlorpromazine, isoniazid, and others (Bakhtiari et al. 2018). Drug‐induced lichenoid reactions are defined by the emergence of lesions like those of lichen planus, a skin and mucosal condition that is marked by chronic inflammation. Following the use of numerous medications (e.g., ACE inhibitors, antibiotics, and anticonvulsants), oral mucosal lesions may develop in the mouth and may result in symptoms such as pain, itching, and sensitivity (Suryana 2020). Medications such as amiodarone, antibiotics, chlorhexidine, and others can produce superficial and temporary mucosal pigmentations (Binmadi et al. 2020).

Within the hard tissue, the most severe and not reversible O‐AEs is medication‐related osteonecrosis of the jaw (MRONJ), associated principally with the use of drugs such as antiresorptive and more rarely with antiangiogenic agents (Wan et al. 2020). This condition can cause the death of the jawbone tissue, resulting in pain and often in the exposure of the bone (Campisi et al. 2020). This is a emerging disease with a huge number of papers each year published in all fields of research from diagnosis to treatment. The risk of MRONJ associated with bisphosphonates and denosumab has been extensively characterized in the literature, and our cases were mainly related to these drugs (Bedogni et al. 2024). However, it appears clear that there was a major under‐reporting of cases, because only 95 ascertained MRONJ cases have been reported in the 3‐year period 2019–2021 to our surveillance system. Such data correspond to about 2.1 cases/year/million population, much lower than data inferable from recent drug surveillance data in Denmark (607 cases reported in 10.5 years in a population of about 5.8 million, corresponding roughly to 9.9 cases/year/million; Ellefsen et al. 2023). Furthermore, the number of cases reported per year in cited three Regions appears very low also in comparison with the number registered in previous years in Piedmont MRONJ case series among patients with cancer (Fusco et al. 2021, 2013) and (in comparison) with MRONJ case series previously published by oral and oncology specialists working in the three participating regions, as mono‐institutional or multicenter studies (Bedogni et al. 2014; Di Fede et al. 2013; Ferlito, Puzzo, and Liardo 2011; Fung et al. 2017; Fusco et al. 2022; Graziani et al. 2012; Oteri et al. 2013; Oteri et al. 2018; Otto et al. 2011; Vescovi et al. 2011). As explained by Parretta et al., in Italy, the first case of MRONJ occurred in 2004 with a peak in 2009, after that, there was a decrease in the reporting. The peak in 2009 was justified by a sollicited reporting which invited healthcare professionals to the report this bisphosphonates‐related safety issue (Jose et al. 2024; Parretta, Sottosanti, et al. 2014). Moreover, in the same year EMA started a review on issues related to the AEs ONJ following the use of bisphosphonates (EMA 2009) and in 2012 bisphosphonates‐induced adverse skeletal events (including ONJ) have been suggested as priorities for drug safety research through meta‐analysis or observational studies (EMA 2013).

To the best of our knowledge, this was the first study conducted in Italy to investigate and to understand AEs at oropharyngeal area by using post‐marketing data from the Italian Pharmacovigilance database (Piedmont, Campania, and Sicily Regions).

The choice to evaluate this specific safety concern was led by the need to examine in‐depth available data on AEs at oropharyngeal area from the real‐life context, which represents a useful point of view to induce better appropriate drug use, healthcare behavior, and more health protection of patients. This is a main strength of our study.

Moreover, several study limitations should be taken into consideration. Firstly, spontaneous monitoring systems are useful as alert tool only because they do not provide accurate epidemiological estimates such as prevalence or incidence of AEs. Moreover, these systems are inclined toward under‐reporting more than over‐reporting of AEs. Moreover, we cannot exclude that there was a distortion of our results from a qualitative and quantitative point of view because of the limited period analyzed, which included also the COVID‐19 pandemic.

In our dataset, it was not possible to establish how many and which ICSRs were reported by dentists because the generic codification “physician/doctor” reported in the RNF for the identification of reporting source included both general practitioners, dental and other medical specialists. However, probably the dentists contributed only in part to the reporting of the ICSRs analyzed. In line with our hypothesis, Ellefsen et al. (2023) found that this professional figure contributed for 19% to the Danish reporting in a 10‐year period analyzed, compared with 44% of physicians. Moreover, they underlined that the reporting of O‐AEs often reflects the opinions emerging from community and professional circles (Ellefsen et al. 2023). This implicates sporadic pattern of AE reporting and not a good sensibilization and a dedicated attitude toward the pharmacovigilance during the clinical practice.

Another study limitation is due to our selection method of the ICSRs based on the type of AEs. Thus, we may not have captured all the reports from dentists in order to analyze their real behavior and contribution in terms of reporting. Moreover, we only focused on O‐AEs, excluding from the analysis all the other AEs (not oropharyngeal) involved in each selected ICSR. As each AE should be seen individually but also in an overview of overall symptoms and signs reported in the ICSRs, mostly, if it is related to allergic or infective conditions, we only touched upon the notoriety of most reported O‐AEs related to the first most reported products.

## 5 Conclusions

The current study provides a descriptive overview of spontaneous reports of oropharyngeal AEs in a part of Italian territory in a 3‐year period. Only 4.1% of ICSRs reported was related to oropharyngeal AEs. Most of the ICSRs were related to COVID‐19 vaccines, probably for the limited time analyzed, which included the first and bigger curve of the COVID‐19 pandemic. For this type of O‐AEs, physicians/doctors seem to be the main reporting source, but we do not know the real contribution of dentists, general practitioners, or other specialists. In agreement with available data in the literature, the results of this post‐marketing analysis suggested an under‐reporting of this type of AEs and the need to train dentists, dental hygienists, and also general practitioners (both in the study courses and in postgraduate training as continuing professional update). The lacking or under‐reporting of AEs by healthcare professionals is a recognized general concern and dentists, such as general practitioners, seem to be no exception. This study is relevant for better reporting of O‐AE in the future and how it contributes to the knowledge of AE and health professionals handling of AE. Given the above‐mentioned limitations of this analysis, pharmacovigilance and observational studies are strongly needed in order to collect more data on the O‐AEs related to drugs and/or vaccines and to investigate better the attitude and the real appeal of dentists.

## Author Contributions

**Liberata Sportiello:** conceptualization, data curation, writing – original draft, formal analysis. **Gaetano La Mantia:** conceptualization, data curation, writing – original draft. **Vera Panzarella:** conceptualization, data curation, writing – original draft. **Giuseppe Colella:** conceptualization, writing – original draft. **Simona Potenza:** supervision, writing – review and editing. **Giacomo Oteri:** conceptualization, writing – review and editing. **Laura Sottosanti:** supervision, writing – review and editing. **Giuseppe Bellavia:** formal analysis, investigation. **Mario Gaio:** formal analysis, investigation. **Alessia Zinzi:** formal analysis, investigation. **Ilaria Morreale:** formal analysis, investigation. **Gaspare Parrinello:** formal analysis, investigation. **Elisabetta Geninatti:** formal analysis, investigation. **Eleonora Marrazzo:** formal analysis, investigation. **Vittorio Fusco:** conceptualization, data curation, writing – review and editing. **Annalisa Capuano:** conceptualization, data curation, supervision, validation, writing – review and editing. **Giuseppina Campisi:** conceptualization, data curation, supervision, writing – review and editing, validation.

## Conflicts of Interest

The authors declare no conflicts of interest.

## Supporting information


Data S1.

## Data Availability

The use of the raw data that support the findings of this study has been authorized by the Italian Medicines Agency (AIFA), which holds the ownership of the data. Data can be available with the permission of AIFA upon reasonable request.
