# Supplementary material for: Oropharyngeal Adverse Events to Drugs and Vaccines: Pharmacovigilance Data From Italy (2019–2021)
Source: Oral Dis. 2024 Oct 6;31(3):993–1005. doi: 10.1111/odi.15145 (PMC12021317; doi:10.1111/odi.15145)
Supplement: Supplementary file 1 — Data S1. [file ODI-31-993-s001.docx]

**Supplementary Table 1. Modified list of all Preferred Terms (PTs) divided in six Standardized MedDRA Query (SMQ) used to perform the data analysis.**

| **Oropharyngeal disorders (SMQ)** |
| --- |
| **Oropharyngeal allergic conditions (SMQ)** |
| Angioedema |
| Gingival swelling |
| Mouth oedema/edema |
| Swelling of tongue |
| Tongue oedema/edema |
| Gingival oedema/Edema gum |
| Palatal oedema/edema |
| Contact stomatitis |
| Idiopathic angioedema |
| Palatal swelling |
| Mouth swelling |
| Pharyngeal oedema |
| Epiglottic oedema/edema |
| Epiglottitis |
| Oropharyngeal swelling |
| Allergic stomatitis |
| **Oropharyngeal conditions (excluding neoplasms. infections and allergies) (SMQ)** |
| Aphthous ulcer |
| Aptyalism |
| Behcet syndrome |
| Buccoglossal syndrome |
| Dry mouth |
| Dry throat |
| Dysphagia |
| Foreign body in pharynx |
| Glossitis |
| Glossodynia |
| Tongue papillary hypertrophy NOS |
| Melkersson- Rosenthal syndrome |
| Mouth bleeding |
| Ulcer buccal |
| Muco-cutaneo-ocular syndrome |
| Odynophagia |
| Oral disorder |
| Oral lichen planus |
| Oral mucosa blister/blistering |
| Oral mucosal/mucosa discolouration |
| Oral mucosal eruption |
| Oral pain/pain oral |
| Oral papule |
| Oral submucosal fibrosis |
| Oropharyngeal spasm |
| Parotid duct obstruction |
| Parotid gland enlargement |
| Plicated tongue |
| Protrusion tongue |
| Ranula |
| Altered saliva |
| Salivary duct obstruction |
| Salivary duct stenosis |
| Salivary gland atrophy |
| Salivary gland calculus |
| Salivary gland enlargement |
| Salivary gland fistula |
| Salivary gland pain |
| Salivary hypersecretion |
| Sjögren's syndrome |
| Stevens-Johnson syndrome |
| Stomatitis |
| Stomatitis haemorrhagic |
| Stomatitis necrotising |
| Stomatitis radiation |
| Submaxillary gland enlargement |
| Throat irritation |
| Throat tightness |
| Tongue blistering |
| Coated tongue/Tongue coated |
| Tongue discoloration/Discoloration tongue |
| Tongue disorder/Tongue disorder NOS |
| Lingua geographica/Geographic tongue/Tongue geographic |
| Tongue hematoma/haematoma |
| Tongue movement disturbance |
| Tongue disorder |
| Tongue rough |
| Tongue spasm/Spasm tongue |
| Tongue ulceration |
| Tonsillar hypertrophy |
| Toxic epidermal necrolysis |
| Parotid lipomatosis |
| Sialectasia |
| Saliva discoloration |
| Ankyloglossia acquired |
| Laceration of mouth |
| Tongue dry |
| Tongue haemorrhage |
| Oral mucosa atrophy |
| Tonsillar ulcer |
| Glossodynia |
| Parotid gland haemorrhage/hemorrhage |
| Mikulicz's syndrome |
| Sialocele |
| Tonsillar atrophy |
| Saburral tongue |
| Uvulitis |
| Tongue eruption |
| Salivary gland cyst |
| Mikulicz's syndrome |
| Palatal disorder |
| Palatal dysplasia |
| Itching mouth |
| Tonsillar disorder |
| Throat lesion |
| Tongue atrophy |
| Lip laceration |
| Salivary duct inflammation |
| Salivary gland mass |
| Oral leukoedema |
| Hypoesthesia oral |
| Paresthesia oral |
| Tonsillar haemorrhage/hemorrhage |
| Oral toxicity |
| Acquired macroglossia |
| Glossoptosis |
| Auriculotemporal syndrome |
| Tongue laceration |
| Salivary gland mucocele/mucocoele |
| Salivary gland disorder/NOS |
| Foaming at mouth |
| Oral mucosal hypertrophy |
| Tonsillolith |
| Angina bullosa haemorrhagica/hemorrhagica |
| Oral mucosal exfoliation |
| Tongue exfoliation |
| Oral mucosa erosion |
| Oroantral fistula |
| Tonsillar inflammation |
| Oral cavity fistula |
| Sialometaplasia |
| Velopharyngeal incompetence |
| Tongue necrosis |
| Oral mucosal erythema |
| Oral disorder |
| Oropharyngeal plaque |
| Oropharyngeal blistering |
| Burning mouth syndrome |
| Oropharyngeal discomfort |
| Oropharyngeal pain |
| Radiation salivary gland injury |
| Atrophic glossitis |
| Tongue pigmentation |
| Tongue itching |
| PFAPA syndrome |
| Chemical burn of oral cavity |
| Oropharyngeal scar |
| Nicotinic stomatitis |
| Pharyngeal dyskinesia |
| Exposed bone in jaw |
| Salivary gland induration |
| Palatal palsy |
| Sialography abnormal |
| Pyostomatitis vegetans |
| Tongue infarction |
| Scalloped tongue |
| Oral mucosa haematoma/hematoma |
| Noninfective sialoadenitis |
| Acute postoperative sialadenitis |
| Coating in mouth |
| Burn oral cavity |
| Cobble stone tongue |
| Traumatic ulcerative granuloma with stromal eosinophilia |
| Oral hyperesthesia |
| Oral blood blister |
| Palatal ulcer |
| Oropharyngeal cobble stone mucosa |
| Oral pigmentation |
| Tongue discomfort |
| Chronic cheek biting |
| MAGIC syndrome |
| Oral contusion |
| Tonsillar erythema |
| Tonsillar exudate |
| Oropharyngeal oedema/edema |
| Tongue erythema |
| Oropharyngeal discolouration/discoloration |
| Transient lingual papillitis |
| Throat clearing |
| Trichoglossia |
| Cricopharyngeal achalasia |
| Pharyngeal dystonia |
| Stiff tongue |
| Palatal polyp |
| Lichenoid dysplasia |
| Tongue thrust |
| Anesthesia oral |
| Lip scab |
| Oral mucosal scab |
| Mucocutaneous disorder |
| SJS-TEN overlap |
| Oral macule |
| Foreign body in pharynx |
| Acute graft versus host disease oral |
| Chronic graft versus host disease oral |
| Oral purpura |
| Oral lichenoid reaction |
| Oral mucosal roughening |
| Oral mucosal scar |
| Tongue induration |
| Palatal prolapse |
| Oropharyngeal lymphoid hyperplasia |
| Acquired soft palate fissure |
| Orofacial granulomatosis |
| Uvula deviation |
| Oral vaccine administration site lesions |
| Pharyngeal hypoesthesia |
| Pharyngeal swelling |
| Pharyngeal erythema |
| Pharyngeal paresthesia |
| Diffuse infiltrative lymphocytosis syndrome |
| **Oropharyngeal infections (SMQ)** |
| Abscess of salivary gland |
| Abscess oral |
| Angina gangrenous |
| Aspergillosis oral |
| Chronic tonsillitis |
| Gingivitis |
| Hand. foot and mouth disease |
| Herpangina |
| Mumps |
| Oral candidiasis |
| Parotid abscess |
| Parotitis |
| Parotiditis |
| Abscess peritonsillar/NOS |
| Sialoadenitis/NOS |
| Tonsillitis/NOS |
| Streptococcal tonsillitis |
| Viral tonsillitis |
| Oral infection |
| Ludwig angina |
| Pharyngotonsillitis |
| Peritonsillitis |
| Oropharyngeal candidiasis |
| Strawberry tongue |
| Gingival abscess |
| Oro-pharyngeal aspergillosis |
| Necrotizing ulcerative gingivostomatitis |
| Necrotizing ulcerative periodontitis |
| Oral pustule |
| Oral fungal infection |
| Oropharyngitis fungal/NOS |
| Oral bacterial infection |
| Oral viral infection |
| Tonsillitis bacterial |
| Tonsillitis fungal |
| Oral helminthic infection |
| Lip infection |
| Oropharyngeal gonococcal infection |
| Tongue abscess |
| Oral herpes |
| Lineal gingival erythema |
| Staphylococcal parotitis |
| Pharyngeal abscess |
| Infective glossitis |
| Viral parotitis |
| Herpes simplex pharyngitis |
| Herpes zoster pharyngitis |
| Tongue fungal infection |
| Oral tuberculosis |
| Bacterial parotitis |
| Parapharyngeal space infection |
| Pharyngitis |
| Bacterial gingivitis |
| **Gingival disorders (SMQ)** |
| Gingival atrophy |
| Gingival bleeding/Bleeding gingival |
| Gingival discolouration/discoloration |
| Gingival disease |
| Erosion gingival |
| Gingival hypertrophy/Gum hypertrophy |
| Gingival hypoplasia/Gum hypoplasia |
| Gingival pain/gum pain/pain gum |
| Gingival/Gum recession |
| Gingivitis ulcerative |
| Periodontal destruction |
| Periodontal disease |
| Gingival injury |
| Gingival blister |
| Itching gum |
| Gingival/Gum ulceration |
| Gingival hyperpigmentation |
| Gingival erythema |
| Periodontal inflammation |
| Noninfective gingivitis |
| Gingival/Gum discomfort |
| Excessive gingival display |
| Gingival/Gum scar |
| Dental root perforation |
| **Oropharyngeal neoplasms (SMQ)** |
| Salivary gland neoplasm benign/NOS |
| Giant cell epulis |
| Gingival polyp |
| Leukoplakia oral/NOS |
| Lip and/or oral cavity cancer recurrent |
| Lip and/or oral cavity cancer stage 0 |
| Lip and/or oral cavity cancer stage I |
| Lip and/or oral cavity cancer stage II |
| Lip and/or oral cavity cancer stage III |
| Lip and/or oral cavity cancer stage IV |
| Malignant palate neoplasm |
| Mouth cyst |
| Oral hairy leukoplakia |
| Oral neoplasm benign |
| Salivary gland cancer recurrent |
| Salivary gland cancer stage 0 |
| Salivary gland cancer stage I |
| Salivary gland cancer stage II |
| Salivary gland cancer stage III |
| Salivary gland cancer stage IV |
| Squamous cell carcinoma of the oral cavity |
| Squamous cell carcinoma of the tongue |
| Tongue neoplasm benign |
| Malignant neoplasm of tongue. unspecified |
| Tonsillar neoplasm |
| Benign tonsillar neoplasm |
| Buccal polyp |
| Salivary gland adenoma |
| Tongue dysplasia |
| Oral fibroma |
| Metastases to mouth |
| Metastases to salivary gland |
| Tongue cyst |
| Gingival cyst |
| Oral cavity cancer metastatic |
| Tongue cancer metastatic |
| Tonsillar cyst |
| Epulis |
| Tongue carcinoma stage 0 |
| Tongue carcinoma stage I |
| Tongue carcinoma stage II |
| Tongue carcinoma stage III |
| Tongue carcinoma stage IV |
| Oral neoplasm/NOS |
| Salivary gland neoplasm/cancer/NOS |
| Lip and/or oral cavity cancer |
| Salivary gland cancer |
| Leukoplakia/NOS |
| Tongue neoplasm/NOS |
| Tonsillar neoplasm/cancer/NOS |
| Metastatic salivary gland cancer |
| Gingival cancer |
| Melanoplakia oral |
| Adenocarcinoma of salivary gland |
| Oral papilloma |
| Tongue cancer recurrent |
| Tonsil cancer metastatic |
| Acinic cell carcinoma of salivary gland |
| Adenoid cystic carcinoma of salivary gland |
| Mucoepidermoid carcinoma of salivary gland |
| Pleomorphic adenoma |
| Papillary cystadenoma lymphomatosum |
| Tongue polyp |
| Carcinoma ex-pleomorphic adenoma |
| Metastases to tonsils |
| Oral haemangioma/hemangioma |
| Oropharyngeal dysplasia |
| Squamous cell carcinoma of the parotid gland |
| Oral melanocytic naevus/nevus |
| HER2 positive salivary gland cancer |
| **Osteonecrosis (SMQ)** |
| Dental necrosis |
| X-ray NOS face and mouth abnormal |
| Jaw lesion excision |
| Jaw pain/Pain in jaw |
| Dental abscess |
| Tooth infection |
| Oral surgery/NOS |
| Abscess jaw |
| Maxillofacial operation |
| Osteonecrosis of jaw |
| Jaw fistula |
| X-ray dental abnormal |
| Osteonecrosis |
| Bone pain/Pain bone |

**Supplementary Table 2. List of all O-AEs in terms of PTs for each SMQ reported in the Individual Case Safety Reports (ICSRs).**

| **SMQ** | **N. PT** | **%** |
| --- | --- | --- |
| **Oropharyngeal conditions (excluding neoplasms. infections and allergies)** | **2192** | **65.9** |
| Oropharyngeal pain | 276 | 8.3 |
| Throat tightness | 266 | 8.0 |
| Paresthesia oral | 256 | 7.7 |
| Dysphagia | 181 | 5.4 |
| Dry mouth | 177 | 5.3 |
| Stomatitis | 159 | 4.8 |
| Aphthous ulcer | 110 | 3.3 |
| Throat irritation | 97 | 2.9 |
| Hypoesthesia oral | 77 | 2.3 |
| Oral disorder | 45 | 1.4 |
| Oropharyngeal discomfort | 41 | 1.2 |
| Stevens-Johnson syndrome | 35 | 1.1 |
| Salivary hypersecretion | 33 | 1.0 |
| Tongue induration | 33 | 1.0 |
| Pharyngeal paresthesia | 31 | 0.9 |
| Tongue discomfort | 30 | 0.9 |
| Itching mouth | 27 | 0.8 |
| Toxic epidermal necrolysis | 26 | 0.8 |
| Glossitis | 21 | 0.6 |
| Dry throat | 17 | 0.5 |
| Odynophagia | 17 | 0.5 |
| Oral pain/pain oral | 17 | 0.5 |
| Tongue itching | 17 | 0.5 |
| Pharyngeal erythema | 17 | 0.5 |
| Mouth bleeding | 16 | 0.5 |
| Glossodynia | 13 | 0.4 |
| Tonsillar hypertrophy | 13 | 0.4 |
| Ulcer buccal | 11 | 0.3 |
| Tongue ulceration | 11 | 0.3 |
| Glossodynia | 9 | 0.3 |
| Tongue papillary hypertrophy | 7 | 0.2 |
| Parotid gland enlargement | 7 | 0.2 |
| Tongue erythema | 7 | 0.2 |
| Oral mucosa blister/blistering | 5 | 0.2 |
| Aptyalism | 5 | 0.2 |
| Oral mucosal eruption | 5 | 0.2 |
| Tongue disorder | 5 | 0.2 |
| Anesthesia oral | 5 | 0.2 |
| Palatal disorder | 4 | 0.1 |
| Tongue discoloration | 4 | 0.1 |
| Tongue haemorrhage | 4 | 0.1 |
| Palatal dysplasia | 3 | 0.1 |
| Plicated tongue | 3 | 0.1 |
| Salivary gland pain | 3 | 0.1 |
| Oropharyngeal oedema | 3 | 0.1 |
| Tonsillar inflammation | 3 | 0.1 |
| Oral mucosal erythema | 3 | 0.1 |
| Pharyngeal swelling | 3 | 0.1 |
| Coated tongue | 3 | 0.1 |
| Burning mouth syndrome | 2 | 0.1 |
| Foaming at mouth | 2 | 0.1 |
| Tongue blistering | 2 | 0.1 |
| Pharyngeal dystonia | 2 | 0.1 |
| Oral disorder | 2 | 0.1 |
| Coating in mouth | 2 | 0.1 |
| Tongue eruption | 2 | 0.1 |
| Pharyngeal hypoesthesia | 2 | 0.1 |
| Geographic tongue/Tongue geographic | 1 | 0.0 |
| Tongue laceration | 1 | 0.0 |
| Oral cavity fistula | 1 | 0.0 |
| Oral contusion | 1 | 0.0 |
| Stomatitis haemorrhagic | 1 | 0.0 |
| Tongue dry | 1 | 0.0 |
| Oral mucosa erosion | 1 | 0.0 |
| Palatal swelling | 1 | 0.0 |
| Salivary gland enlargement | 1 | 0.0 |
| Tongue haematoma | 1 | 0.0 |
| Tongue pigmentation | 1 | 0.0 |
| Lip laceration | 1 | 0.0 |
| Laceration of mouth | 1 | 0.0 |
| Foreign body in pharynx | 1 | 0.0 |
| Oropharyngeal plaque | 1 | 0.0 |
| **Oropharyngeal allergic conditions** | **453** | **13.6** |
| Angioedema | 166 | 5.0 |
| Tongue oedema | 74 | 2.2 |
| Swelling of tongue | 71 | 2.1 |
| Palatal oedema | 47 | 1.4 |
| Pharyngeal oedema | 29 | 0.9 |
| Mouth oedema | 17 | 0.5 |
| Gingival swelling | 13 | 0.4 |
| Mouth swelling | 11 | 0.3 |
| Gingival oedema | 10 | 0.3 |
| Palatal swelling | 4 | 0.1 |
| Oropharyngeal swelling | 4 | 0.1 |
| Epiglottic oedema | 3 | 0.1 |
| Itching mouth | 2 | 0.1 |
| Tongue itching | 2 | 0.1 |
| **Osteonecrosis** | **362** | **10.9** |
| Bone pain | 177 | 5.3 |
| Osteonecrosis of jaw | 95 | 2.9 |
| Osteonecrosis | 28 | 0.8 |
| Jaw pain | 33 | 1.0 |
| Dental abscess | 17 | 0.5 |
| Jaw fistula | 11 | 0.3 |
| Abscess oral | 1 | 0.0 |
| **Oropharyngeal infections** | **207** | **6.2** |
| Oral herpes | 109 | 3.3 |
| Gingivitis | 29 | 0.9 |
| Pharyngitis | 27 | 0.8 |
| Oral candidiasis | 14 | 0.4 |
| Tonsillitis | 6 | 0.2 |
| Oral fungal infection | 4 | 0.1 |
| Pharyngotonsillitis | 4 | 0.1 |
| Abscess oral | 3 | 0.1 |
| Gingival abscess | 3 | 0.1 |
| Pharyngitis bacterial | 2 | 0.1 |
| Sialoadenitis | 1 | 0.0 |
| Tooth infection | 1 | 0.0 |
| Infective glossitis | 1 | 0.0 |
| Epiglottitis | 1 | 0.0 |
| Parotiditis | 1 | 0.0 |
| Oral infection | 1 | 0.0 |
| **Gingival disorders** | **106** | **3.2** |
| Gingival bleeding | 67 | 2.0 |
| Gingival pain | 10 | 0.3 |
| Noninfective gingivitis | 9 | 0.3 |
| Gingival hypertrophy | 5 | 0.2 |
| Gingival discomfort | 4 | 0.1 |
| Gingivitis | 4 | 0.1 |
| Gingival erythema | 3 | 0.1 |
| Gingival recession | 2 | 0.1 |
| Gingival blister | 1 | 0.0 |
| Gingival ulceration | 1 | 0.0 |
| **Oropharyngeal neoplasms** | **4** | **0.1** |
| Leukoplakia oral | 2 | 0.1 |
| Oral neoplasm | 1 | 0.0 |
| Oral papilloma | 1 | 0.0 |
| **Total** | **3324** | **100.0** |

**Supplementary Table 3. Frequency of suspected medicinal products for each therapeutic group (2nd level - Anatomical Therapeutic Chemical, ATC).**

| **2nd level ATC** | **Suspected medicinal product** | **N.** | **%** |
| --- | --- | --- | --- |
| J01 (N=188) | Amoxicillin/clavulanic acid | 51 | 27.1% |
|  | Ceftriaxone | 16 | 8.5% |
|  | Meropenem | 15 | 8.0% |
|  | Ciprofloxacin | 10 | 5.3% |
|  | Clarithromycin | 10 | 5.3% |
|  | Levofloxacin | 10 | 5.3% |
|  | Amoxicillin | 9 | 4.8% |
|  | Azithromycin | 8 | 4.3% |
|  | Vancomycin | 7 | 3.7% |
|  | Cefixime | 6 | 3.2% |
|  | Piperacillin/tazobactam | 6 | 3.2% |
|  | Fosfomicin | 4 | 2.1% |
|  | Trimethoprim/sulfamethoxazole | 3 | 1.6% |
|  | Amikacin | 2 | 1.1% |
|  | Bacampicillin | 2 | 1.1% |
|  | Cefpodoxime | 2 | 1.1% |
|  | Ceftazidime | 2 | 1.1% |
|  | Ceftobiprole | 2 | 1.1% |
|  | Daptomycin | 2 | 1.1% |
|  | Lincomycin | 2 | 1.1% |
|  | Netilmicin | 2 | 1.1% |
|  | Prulifloxacin | 2 | 1.1% |
|  | Sulfamethoxazole/trimetoprim | 2 | 1.1% |
|  | Acetoxyethylcefuroxime | 1 | 0.5% |
|  | Benzathine Penicillin G | 1 | 0.5% |
|  | Cefodizime | 1 | 0.5% |
|  | Cefotaxime | 1 | 0.5% |
|  | Clindamycin | 1 | 0.5% |
|  | Clindamycin, combinations | 1 | 0.5% |
|  | Dalbavancin | 1 | 0.5% |
|  | Doxycycline | 1 | 0.5% |
|  | Ertapenem | 1 | 0.5% |
|  | Linezolid | 1 | 0.5% |
|  | Lymecycline | 1 | 0.5% |
|  | Roxithromycin | 1 | 0.5% |
|  | Teicoplanin | 1 | 0.5% |
| J02 (N=4) | Amphotericin B | 1 | 25.0% |
|  | Anidulafungin | 1 | 25.0% |
|  | Flunisolide | 1 | 25.0% |
|  | Voriconazole | 1 | 25.0% |
| J04 (N=1) | Rifampicine | 1 | 100.0% |
| J05 (N=10) | Aciclovir | 4 | 40.0% |
|  | Lamivudine | 3 | 30.0% |
|  | Sofosbuvir/velpatasvir | 2 | 20.0% |
|  | Emtricitabine, tenofovir alafenamide, elvitegravir e cobicistat | 1 | 10.0% |
| J06 (N=3) | Intravenous immunoglobulin | 3 | 100.0% |
| J07 (1528) | mRNA COVID-19 Vaccine | 1203 | 78.7% |
|  | Viral vector-bases COVID-19 vaccine | 212 | 13.9% |
|  | Meningococcal vaccine | 35 | 2.3% |
|  | Measles, mumps, rubella and varicella vaccine | 24 | 1.6% |
|  | Measles, mumps and rubella vaccine | 19 | 1.2% |
|  | Seasonal influenza vaccine | 12 | 0.8% |
|  | Pneumococcal vaccine | 9 | 0.6% |
|  | Diphtheria, tetanus, pertussis, hepatitis B, poliomyelitis and Haemophilus influenzae type-b conjugate vaccine | 8 | 0.5% |
|  | Rotavirus vaccine | 2 | 0.1% |
|  | Diphtheria, pertussis, tetanus vaccine | 1 | 0.1% |
|  | Hepatitis B vaccine | 1 | 0.1% |
|  | Human Papillomavirus vaccine | 1 | 0.1% |
|  | Vaccine for tuberculosis | 1 | 0.1% |
| L01 (N=633) | Fluorouracil | 83 | 13.1% |
|  | Oxaliplatin | 57 | 9.0% |
|  | Paclitaxel | 42 | 6.6% |
|  | Methotrexate | 37 | 5.8% |
|  | Rituximab | 34 | 5.4% |
|  | Carboplatin | 31 | 4.9% |
|  | Cyclophosfamide | 28 | 4.4% |
|  | Bevacizumab | 20 | 3.2% |
|  | Docetaxel | 20 | 3.2% |
|  | Irinotecan | 20 | 3.2% |
|  | Trastuzumab | 19 | 3.0% |
|  | Vincristine | 16 | 2.5% |
|  | Cisplatin | 13 | 2.1% |
|  | Doxorubicin | 13 | 2.1% |
|  | Etoposide | 12 | 1.9% |
|  | Epirubicin | 11 | 1.7% |
|  | Nivolumab | 11 | 1.7% |
|  | Panitumumab | 11 | 1.7% |
|  | Sorafenib | 11 | 1.7% |
|  | Everolimus | 10 | 1.6% |
|  | Pegaspargase | 9 | 1.4% |
|  | Daunorubicin | 8 | 1.3% |
|  | Pembrolizumab | 8 | 1.3% |
|  | Bendamustine | 6 | 0.9% |
|  | Pertuzumab | 6 | 0.9% |
|  | Cetuximab | 5 | 0.8% |
|  | Gemcitabine | 5 | 0.8% |
|  | Imatinib | 5 | 0.8% |
|  | Pemetrexed | 5 | 0.8% |
|  | Sunitinib | 5 | 0.8% |
|  | Capecitabine | 4 | 0.6% |
|  | Citarabina | 4 | 0.6% |
|  | Regorafenib | 4 | 0.6% |
|  | Afatinib | 3 | 0.5% |
|  | Cabozantinib | 3 | 0.5% |
|  | Daratumumab | 3 | 0.5% |
|  | Ifosfamide | 3 | 0.5% |
|  | Nintedanib | 3 | 0.5% |
|  | Osimertinib | 3 | 0.5% |
|  | Palbociclib | 3 | 0.5% |
|  | Vinorelbine | 3 | 0.5% |
|  | Alectinib | 2 | 0.3% |
|  | Arsenic trioxide | 2 | 0.3% |
|  | Brentuximab vedotin | 2 | 0.3% |
|  | Dabrafenib | 2 | 0.3% |
|  | Ibrutinib | 2 | 0.3% |
|  | Mercaptopurine | 2 | 0.3% |
|  | Temozolomide | 2 | 0.3% |
|  | Thiotepa | 2 | 0.3% |
|  | Abemaciclib | 1 | 0.2% |
|  | Adriblastine | 1 | 0.2% |
|  | Azacitidine | 1 | 0.2% |
|  | Binimetinib | 1 | 0.2% |
|  | Cabazitaxel | 1 | 0.2% |
|  | Carfilzomib | 1 | 0.2% |
|  | Cemiplimab | 1 | 0.2% |
|  | Cladribine | 1 | 0.2% |
|  | Decitabine | 1 | 0.2% |
|  | Encorafenib | 1 | 0.2% |
|  | Farmorubicin | 1 | 0.2% |
|  | Ipilimumab | 1 | 0.2% |
|  | Mitomycin | 1 | 0.2% |
|  | Pazopanib | 1 | 0.2% |
|  | Ribociclib | 1 | 0.2% |
|  | Trametinib | 1 | 0.2% |
|  | Trifluridine, combinations | 1 | 0.2% |
|  | Venetoclax | 1 | 0.2% |
|  | Vinblastine | 1 | 0.2% |
|  | Vindesine | 1 | 0.2% |
| L02 (N=7) | Fulvestrant | 2 | 28.6% |
|  | Triptorelin | 2 | 28.6% |
|  | Bicalutamide | 1 | 14.3% |
|  | Degarelix | 1 | 14.3% |
|  | Letrozole | 1 | 14.3% |
| L03 (N=16) | Interferon beta | 6 | 37.5% |
|  | Glatiramer | 5 | 31.3% |
|  | Filgrastim | 2 | 12.5% |
|  | Lenograstim | 2 | 12.5% |
|  | Pegfilgrastim | 1 | 6.3% |
| L04 (N=127) | Adalimumab | 29 | 22.8% |
|  | Infliximab | 11 | 8.7% |
|  | Mycophenolic acid | 10 | 7.9% |
|  | Golimumab | 9 | 7.1% |
|  | Abatacept | 7 | 5.5% |
|  | Ocrelizumab | 7 | 5.5% |
|  | Secukinumab | 7 | 5.5% |
|  | Baricitinib | 6 | 4.7% |
|  | Natalizumab | 6 | 4.7% |
|  | Ustekinumab | 5 | 3.9% |
|  | Vedolizumab | 5 | 3.9% |
|  | Etanercept | 4 | 3.1% |
|  | Azathioprine | 2 | 1.6% |
|  | Certolizumab | 2 | 1.6% |
|  | Fingolimod | 2 | 1.6% |
|  | Leflunomide | 2 | 1.6% |
|  | Sarilumab | 2 | 1.6% |
|  | Tofacitinib | 2 | 1.6% |
|  | Upadacitinib | 2 | 1.6% |
|  | Canakinumab | 1 | 0.8% |
|  | Dimethyl fumarate | 1 | 0.8% |
|  | Guselkumab | 1 | 0.8% |
|  | Ixekizumab | 1 | 0.8% |
|  | Lenalidomide | 1 | 0.8% |
|  | Pirfenidone | 1 | 0.8% |
|  | Tocilizumab | 1 | 0.8% |
| M01 (N=79) | Ketoprofen | 33 | 41.8% |
|  | Ibuprofen | 20 | 25.3% |
|  | Etoricoxib | 6 | 7.6% |
|  | Nimesulide | 5 | 6.3% |
|  | Ketorolac | 4 | 5.1% |
|  | Meloxicam | 3 | 3.8% |
|  | Naproxen | 2 | 2.5% |
|  | Piroxicam | 2 | 2.5% |
|  | Celecoxib | 1 | 1.3% |
|  | Condroitin | 1 | 1.3% |
|  | Indometacin/Caffeine/Prochlorperazine | 1 | 1.3% |
|  | Niflumic acid | 1 | 1.3% |
| M02 (N=14) | Diclofenac | 14 | 100.0% |
| M03 (N=11) | Tiocolchicoside | 4 | 36.4% |
|  | Baclofene | 3 | 27.3% |
|  | Tizanidine | 3 | 27.3% |
|  | Cyclobenzaprine | 1 | 9.1% |
| M04 (N=23) | Allopurinol | 22 | 95.7% |
|  | Febuxostat | 1 | 4.3% |
| M05 (N=144) | Denosumab | 60 | 41.7% |
|  | Zoledronic acid | 38 | 26.4% |
|  | Alendronic acid | 22 | 15.3% |
|  | Ibandronic acid | 7 | 4.9% |
|  | Alendronic acid/colecalciferol | 5 | 3.5% |
|  | Pamidronic acid | 4 | 2.8% |
|  | Clodronic acid | 3 | 2.1% |
|  | Risedronic acid | 3 | 2.1% |
|  | Risedronatic acid | 2 | 1.4% |
| M09 (N=1) | Hyaluronic acid | 1 | 100.0% |
| N01 (N=1) | Lidocaine/cetrimide | 1 | 100.0% |
| N02 (N=62) | Acetylsalicylic acid | 25 | 40.3% |
|  | Paracetamol | 19 | 30.6% |
|  | Tramadol | 6 | 9.7% |
|  | Ossicodone (oxycodone)/naloxone | 3 | 4.8% |
|  | Delta-9-tetrahydrocannabinol/cannabidiol | 1 | 1.6% |
|  | Frovatriptan | 1 | 1.6% |
|  | Metamizole | 1 | 1.6% |
|  | Oxycodone/paracetamol | 1 | 1.6% |
|  | Paracetamol/ascorbic acid/propyphenazone | 1 | 1.6% |
|  | Paracetamol/codeine | 1 | 1.6% |
|  | Tapentadol | 1 | 1.6% |
|  | Tramadol/paracetamol | 1 | 1.6% |
|  | Zolmitriptan | 1 | 1.6% |
| N03 (N=16) | Pregabalin | 5 | 31.3% |
|  | Carbamazepine | 2 | 12.5% |
|  | Lacosamide | 2 | 12.5% |
|  | Lamotrigine | 2 | 12.5% |
|  | Valproic acid | 2 | 12.5% |
|  | Oxcarbazepine | 1 | 6.3% |
|  | Phenobarbital | 1 | 6.3% |
|  | Phenytoin | 1 | 6.3% |
| N04 (N=1) | Rasagiline | 1 | 100.0% |
| N05 (N=31) | Aripiprazole | 8 | 25.8% |
|  | Haloperidol | 7 | 22.6% |
|  | Quetiapine | 6 | 19.4% |
|  | Risperidone | 3 | 9.7% |
|  | Triazolam | 2 | 6.5% |
|  | Clozapine | 1 | 3.2% |
|  | Delorazepam | 1 | 3.2% |
|  | Lorazepam | 1 | 3.2% |
|  | Olanzapine | 1 | 3.2% |
|  | Promazine | 1 | 3.2% |
| N06 (N=12) | Amitriptyline | 4 | 33.3% |
|  | Duloxetine | 2 | 16.7% |
|  | Escitalopram | 2 | 16.7% |
|  | Levo-acetylcarnitine | 2 | 16.7% |
|  | Clomipramine | 1 | 8.3% |
|  | Sertraline | 1 | 8.3% |
| N07 (N=2) | Cinnarizine | 2 | 100.0% |
| B01 (N=65) | Clopidogrel | 13 | 20.0% |
|  | Rivaroxaban | 13 | 20.0% |
|  | Warfarin | 10 | 15.4% |
|  | Apixaban | 6 | 9.2% |
|  | Edoxaban | 4 | 6.2% |
|  | Ticagrelor | 4 | 6.2% |
|  | Dabigatran | 4 | 6.2% |
|  | Enoxaparin | 2 | 3.1% |
|  | Selexipag | 2 | 3.1% |
|  | Acenocumarol | 1 | 1.5% |
|  | Alteplase | 1 | 1.5% |
|  | Bemiparin | 1 | 1.5% |
|  | Clopidogrel/acetylsalicylic acid | 1 | 1.5% |
|  | Endoxaban | 1 | 1.5% |
|  | Fondaparinux | 1 | 1.5% |
|  | Ticlopidine | 1 | 1.5% |
| B02 (N=2) | Emicizumab | 1 | 50.0% |
|  | Vitamin K | 1 | 50.0% |
| B03 (N=6) | Ferric carboxymaltose | 2 | 33.3% |
|  | Folic acid | 2 | 33.3% |
|  | Iron | 1 | 16.7% |
|  | Luspatercept | 1 | 16.7% |
| B05 (N=1) | Sodium | 1 | 100.0% |
| C01 (N=10) | Ivabradine | 5 | 50.0% |
|  | Adenosine | 1 | 10.0% |
|  | Amiodarone | 1 | 10.0% |
|  | Isosorbide dinitrate | 1 | 10.0% |
|  | Lidocaine | 1 | 10.0% |
|  | Ranolazine | 1 | 10.0% |
| C02 (N=2) | Clonidine | 1 | 50.0% |
|  | Terazosin | 1 | 50.0% |
| C03 (N=1) | Furosemide | 1 | 100.0% |
| C04 (N=1) | Diltiazem | 1 | 100.0% |
| C07 (N=4) | Nebivolol | 2 | 50.0% |
|  | Atenolol | 1 | 25.0% |
|  | Nebivolol/hydrochlorothiazide | 1 | 25.0% |
| C08 (N=3) | Amlodipine | 1 | 33.3% |
|  | Lercanidipine | 1 | 33.3% |
|  | Nifedipine | 1 | 33.3% |
| C09 (N=24) | Ramipril | 10 | 41.7% |
|  | Losartan | 3 | 12.5% |
|  | Enalapril | 2 | 8.3% |
|  | Ramipril/amlodipine | 2 | 8.3% |
|  | Candesartan and diuretics | 1 | 4.2% |
|  | Irbesartan/hydrochlorothiazide | 1 | 4.2% |
|  | Olmesartan medoxomil | 1 | 4.2% |
|  | Olmesartan medoxomil/hydrochlorothiazide | 1 | 4.2% |
|  | Perindopril/amlodipine | 1 | 4.2% |
|  | Ramipril and diuretics | 1 | 4.2% |
|  | Telmisartan and diuretics | 1 | 4.2% |
| C10 (N=11) | Ezetimibe | 4 | 36.4% |
|  | Atorvastatin | 2 | 18.2% |
|  | Rosuvastatin | 2 | 18.2% |
|  | Alirocumab | 1 | 9.1% |
|  | Lovastatin | 1 | 9.1% |
|  | Simvastatin | 1 | 9.1% |
| V01 (N=15) | Gramineous pollen | 15 | 100.0% |
| V03 (N=7) | Mesna | 6 | 85.7% |
|  | Deferasirox | 1 | 14.3% |
| V08 (N=25) | Iopromide | 10 | 40.0% |
|  | Iomeprol | 8 | 32.0% |
|  | Gadoteric acid | 3 | 12.0% |
|  | Gadobutrol | 2 | 8.0% |
|  | Iobitridol | 1 | 4.0% |
|  | Ioexol | 1 | 4.0% |
| V09 (N=1) | Tetrofosmin | 1 | 100.0% |
| V10 (N=1) | Radium-dichloride (223RA23) | 1 | 100.0% |
| A01 (N=1) | Daktarin | 1 | 100.0% |
| A02 (N=12) | Pantoprazole | 5 | 41.7% |
|  | Esomeprazole | 3 | 25.0% |
|  | Bismuth subcitrate potassium/metronidazole/tetracycline | 2 | 16.7% |
|  | Lansoprazol | 1 | 8.3% |
|  | Omeprazole | 1 | 8.3% |
| A03 (N=5) | Floroglucinol | 1 | 20.0% |
|  | Fluconazole | 1 | 20.0% |
|  | Gefitinib | 1 | 20.0% |
|  | Metoclopramide | 1 | 20.0% |
|  | Trimebutine/medazepam | 1 | 20.0% |
| A04 (N=1) | Aprepitant | 1 | 100.0% |
| A06 (N=3) | Bisacodyl | 1 | 33.3% |
|  | Macrogol 3350/sodium bicarbonate/sodium chloride/potassium chloride | 1 | 33.3% |
|  | Macrogol 4000 | 1 | 33.3% |
| A07 (N=9) | Betamethasone | 5 | 55.6% |
|  | Loperamide | 2 | 22.2% |
|  | Mesalazin | 1 | 11.1% |
|  | Sulfasalazine | 1 | 11.1% |
| A10 (N=7) | Semaglutide | 2 | 28.6% |
|  | Alogliptin | 1 | 14.3% |
|  | Canagliflozin/metformin | 1 | 14.3% |
|  | Dapagliflozin | 1 | 14.3% |
|  | Metformin | 1 | 14.3% |
|  | Pioglitazone | 1 | 14.3% |
| A11 (N=1) | Cholecalciferol | 1 | 100.0% |
| A16 (N=1) | Adenosylmethionine | 1 | 100.0% |
| R01 (N=1) | Naphazoline | 1 | 100.0% |
| R02 (N=3) | Alcool benzilico/sodio benzoato | 1 | 33.3% |
|  | Benzydamine | 1 | 33.3% |
|  | Dichlorophenyl carbinol/sodium benzoate | 1 | 33.3% |
| R03 (N=14) | Tiotropium bromide | 2 | 14.3% |
|  | Beclometasone | 1 | 7.1% |
|  | Beclometasone/formoterol | 1 | 7.1% |
|  | Benralizumab | 1 | 7.1% |
|  | Flurbiprofene | 1 | 7.1% |
|  | Formoterol and beclometasone | 1 | 7.1% |
|  | Glycopyrronium bromide | 1 | 7.1% |
|  | Mepolizumab | 1 | 7.1% |
|  | Montelukast | 1 | 7.1% |
|  | Omalizumab | 1 | 7.1% |
|  | Salbutamol/ipratropium bromide | 1 | 7.1% |
|  | Vilanterol and fluticasone furoate | 1 | 7.1% |
|  | Vilanterol, umeclidinium bromide and fluticasone furoate | 1 | 7.1% |
| R05 (N=6) | Acetylcysteine | 2 | 33.3% |
|  | Bromhexine | 1 | 16.7% |
|  | Dextromethorphan/guaifenesin | 1 | 16.7% |
|  | Dextromethorphane | 1 | 16.7% |
|  | Pyridinol | 1 | 16.7% |
| R06 (N=7) | Ebastine | 3 | 42.9% |
|  | Cetirizine | 2 | 28.6% |
|  | Bilastine | 1 | 14.3% |
|  | Levocetirizine | 1 | 14.3% |
| R07 (N=2) | Ivacaftor | 1 | 50.0% |
|  | Tezacaftor/ivacaftor | 1 | 50.0% |
| H02 (N=11) | Prednisone | 4 | 36.4% |
|  | Desametasone | 4 | 36.4% |
|  | Methylprednisolone | 3 | 27.3% |
| H03 (N=1) | Levothyroxine | 1 | 100.0% |
| H05 (N=1) | Teriparatide | 1 | 100.0% |
| D01 (N=2) | Bifonazole | 1 | 50.0% |
|  | Terbinafine | 1 | 50.0% |
| D08 (N=1) | Tosylchloramide | 1 | 100.0% |
| D10 (N=2) | Adapalene benzoyl peroxide | 1 | 50.0% |
|  | Benzoyl Peroxide | 1 | 50.0% |
| D11 (N=3) | Dupilumab | 3 | 100.0% |
| G03 (N=2) | Progesterone | 1 | 50.0% |
|  | Ulipristal | 1 | 50.0% |
| G04 (N=6) | Tadalafil | 2 | 33.3% |
|  | Alfuzosin | 1 | 16.7% |
|  | Flavoxate and propifenazone | 1 | 16.7% |
|  | Oxybutynin | 1 | 16.7% |
|  | Tamsulosin | 1 | 16.7% |
| P01 (N=2) | Atovaquone/proguanil | 1 | 50.0% |
|  | Hydroxychloroquine | 1 | 50.0% |
| P02 (N=1) | Mebendazole | 1 | 100.0% |
| S01 (N=2) | Aflibercept | 1 | 50.0% |
|  | Homatropine | 1 | 50.0% |

J01 Antibacterials for systemic use, J02 Antimycotics for systemic use, J04 Antimycobacterials, J05 Antivirals for systemic use, J06 Immune sera and immunoglobulins, J07 Vaccines, L01 Antineoplastic agents, L02 Endocrine therapy, L03 Immunostimulants, L04 Immunosuppressants, M01 AntiInflammatory and antirheumatic products, M02 Topical products for joint and muscular pain, M03 Muscle relaxants, M04 Antigout preparations, M05 Drugs for treatment of bone diseases, M09 Other drugs for disorders of the musculo-skeletal system, N01 Anesthetics, N02 Analgesics, N03 Antiepileptics, N04 Anti-parkinson drugs, N05 Psycholeptics, N06 Psychoanaleptics, N07 Other nervous system drugs, B01 Antithrombotic agents, B02 Antihemorrhagics, B03 Antianemic preparations, B05 Blood substitutes and perfusion solutions, C01 Cardiac therapy, C02 Antihypertensives, C03 Diuretics, C04 Peripheral vasodilators, C07 Beta blocking agents, C08 Calcium channel blockers, C09 Agents acting on the renin-angiotensin system, C10 Lipid modifying agents, V01 Allergens, V03 All other therapeutic products, V08 Contrast media, V09 Diagnostic radiopharmaceuticals, V10 Therapeutic radiopharmaceuticals, A01 stomatological preparations, A02 drugs for acid related disorders, A03 drugs for functional gastrointestinal disorders, A04 antiemetics and antinauseants, A06 Drugs for constipation, A07 Antidiarrheals, intestinal antiinflammatory/antiinfective agents, A10 Drugs used in diabetes, A11 Vitamins, A16 Other alimentary tract and metabolism products, R01 Nasal preparations, R02 Throat preparations, R03 Drugs for obstructive airway diseases, R05 Cough and cold preparations, R06 Antihistamines for systemic use, R07 Other respiratory system products, H02 Corticosteroids for systemic use, H03 Thyroid therapy, H05 Calcium homeostasis, D01 Antifungals for dermatological use, D08 Antiseptics and disinfectants, D10 Anti-acne preparations, D11 Other dermatological preparations, G03 Sex hormones and modulators of the genital system, G04 Urologicals, P01 Antiprotozoals, P02 Anthelmintics, S01 Ophthalmologicals.
